# Modeling anesthesia medication delivery using the SEIPS 101 tools

**DOI:** 10.1016/j.apergo.2025.104555

**Published:** 2025-05-22

**Authors:** Elise DeForest, Ken Catchpole, Connor Lusk, James H. Abernathy, David M. Neyens

**Affiliations:** a College of Medicine, Medical University of South Carolina, Charleston, SC, USA; b Department of Anesthesia and Perioperative Medicine, Medical University of South Carolina, Charleston, SC, USA; c Department of Anesthesiology and Critical Care Medicine, Johns Hopkins University, Baltimore, MD, USA; d Department of Industrial Engineering, Department of Bioengineering, Clemson University, Clemson, SC, USA

**Keywords:** Anesthesia medication delivery, Human factors, SEIPS, Systems safety, SEIPS 101

## Abstract

*Background:* Reducing the risk of patient harm during anesthesia medication administration in perioperative settings has been a long-term goal in patient safety. SEIPS 101 tools, provide a series of practice-orientated techniques to apply systems model in real clinical practice, potentially offering a straightforward approach to mapping perioperative medication delivery systems. Data was collected during direct observations of thirty-eight anesthetics, totaling over 100 h on anesthesia providers’ common tasks and interactions with people, environments, tools, and technologies. Observation data, notes, interviews, and literature were organized to create six SEIPS 101 tools demonstrating the complexity of anesthesia medication delivery. The *Anesthesia PETT Scan* represents the facilitators and barriers associated with differences in individual expertise, preferences, and potential conflict between providers. *The People Map* demonstrates the wide range of relevant individuals in medication delivery. *The Task x Tools Matrix* depicts the broad range of interconnected processes to provide anesthesia. *The Journey Map* describes the path used to deliver a medication. *The Anesthesia Work System Interactions Map* identifies necessary interactions that providers have with tools, tasks, people, and environment for successful anesthetics. *The Outcome Matrix* describes various stakeholder experiences and outcomes that contribute to overall system complexity. Identifying and describing the complexity in the anesthesia care delivery system is critical for effective and efficient process-centric interventions. This systems analysis may increase awareness to the limitations of current approaches and improve upon methods and interventions for understanding errors, safety, and the nature of clinical expertise and decision making.

## 1. Introduction

Reducing the risk of patient harm during anesthesia medication administration in perioperative settings has been a long-term goal in patient safety. Medication safety incidents – such as delivering the wrong drug, dose, concentration, route or mislabeling – remain relatively common (Nanji, 2020). Individual errors, which may lead to harm in certain contexts, occur in the administration of as many as one in twenty perioperative medications, with around 80 % considered preventable (Nanji et al., 2016). A variety of interventions have been suggested (Wahr et al., 2017), mostly concerned with tools and technology (e.g., barcode scanners, clinical decision support, smart infusion pumps), standardization (of labels, concentrations, medication storage and prefilled syringes), and better communication and teamwork (to improve situational awareness, coordination, and cross-checking) (Wahr et al., 2017; Nanji, 2020). However, few interventions have had widespread success in reducing medication harm (Jensen et al., 2004; Wahr et al., 2017), and only one randomized controlled interventional trial has been performed (Merry et al., 2011). A more sophisticated, comprehensive understanding of medication delivery systems may offer insights into successful intervention and implementation approaches.

Systems engineering analysis techniques can offer new ways to understand the perioperative drug delivery process, leading to new approaches to intervention (Cooper et al., 2019). Both Failure Modes Effects Analysis (FMEA) (Martin et al., 2017) and Systems-Theoretic Process Analysis (STPA) (Samost-Williams and Nanji, 2020) have been applied to medication delivery before. While FMEA can be useful when exploring individual component failures in known, linear, engineered systems (Leveson and Stephanopoulos, 2013), or interactions between a device and human (Carayon, 2012), it may not effectively account for adaptations, local individual variation, or non-linear effects in broader systems. A prior STPA analysis recommended double checks at the frontline, formalized communication channels when making management decisions for patient care, and changes to supplies and room design at the leadership level (Samost-Williams and Nanji, 2020). Rather than focusing on errors or harm events alone, a rich an nuanced perspective can also be developed by exploring more generally how systems-of-work function to successfully every day achieve their goals.

The Systems Engineering Initiative for Patient Safety (SEIPS) model (Carayon et al. 2006, 2020; Holden et al., 2013), has become one of the most well used models for healthcare systems safety but has yet to be applied to the perioperative medication delivery process. Conceptually, it represents the interconnectedness of and interactions between the people, tools and technology, tasks, and environment within a healthcare system (Carayon et al. 2006, 2020; Holden et al., 2013). It has supported successful intervention design and implementation, most notably in outpatient surgery, cardiac surgical care, intensive care units and nursing homes (Carayon et al., 2005; Holden et al., 2013). The recent publication of the SEIPS 101 tools (Holden and Carayon, 2021) developed seven simple tools that offer a relatively straightforward approach to mapping clinical systems. SEIPS 101’s People, Environment, Tools and Tasks (PETT) Scan models the various facilitators and barriers. The People Map explores the range of relevant individuals involved. The Task x Tools Matrix depicts the various jobs and equipment used. The Journey Map describes the general path of the patient. The Anesthesia Work System Interactions Map identifies the interactions necessary. The Outcome Matrix describes the various stakeholder experiences and outcomes.

The objective of this study was to use the SEIPS 101 tools to describe the anesthesia and perioperative medication delivery system. In doing so, we hoped to explore the practical value of the SEIPS 101 tools for understanding and improving healthcare delivery from a systems perspective, to describe the hidden complexities of anesthesia work and to identify potential interventions that might improve anesthesia medication safety.

## 2. Methods

### 2.1. Setting

This study was conducted at an academic hospital system in the Southeastern United States comprised of 903 patient beds and 41 operating rooms (ORs) under Institutional Review Board approval (IRB# Pro00082024). This academic hospital system in a public metropolitan area. This institution practices through an anesthesia care team model in which in-room anesthesia providers (IRAP) including certified registered nurse anesthetists (CRNAs) and fellow and resident anesthesiologists provide anesthesia care under the supervision of a supervising anesthesia provider (SAP) who are typically attending anesthesiologists (Glance, 2000; Hyder et al., 2016). The institution also practices with a pharmacy-based model in which the hospital OR pharmacy supplies and prepares the medications, while anesthesia providers dilute, reconstitute, and draw the medications in the ORs.

### 2.2. Data collection

Data was collected until information saturation was reached. Thirty-eight surgical cases requiring anesthetics, totaling over 100 h, were observed. The procedures were sampled on a convenience basis. They were observed at different times of the day (morning and afternoons) and different days of the week (weekdays and weekends), and included several different specialties (general surgery, orthopedic, robotic, otolaryngology and obstetric cases) and urgencies (planned and emergent). Two research assistants (ED, GS) completed direct observations and data collection, all trained with a basic understanding of human factors and systems engineering principles and methods, including the SEIPS model and SEIPS 101 tools. For each case, observers used a structured table that prompted them to document specific observations centered around the SEIPS factors including as the anesthesia providers’ common tasks and their interactions with other people, environments, tools, and technologies from the preoperative to postoperative phase. Observers also conducted observations of clinical work as it happened recording fieldnotes along with in-situ interviews with providers. In-situ interviews entailed clarifying questions to providers during observations such as their typical responsibilities and daily tasks, facilitators and barriers of the occupation, function of each tool, etc.

### 2.3. Model development

The analysis broadly focused on medication delivery and its interactions with other anesthesia tasks and systems but did not focus on all aspects of delivery and patient safety, nor only on medication delivery. One research assistant compiled and systematically organized the observation data, notes, interviews, and a literature evidence base including the SEIPS model (Carayon et al., 2006), SEIPS 2.0 model (Holden et al., 2013), SEIPS 3.0 model (Carayon et al., 2020), and SEIPS 101 tools (Holden and Carayon, 2021) to create six tools demonstrating the complexity of the anesthesia medication delivery system. Two human factors researchers (KC and CL), one systems engineer (DN), two attending anesthesiologists (JA, CT), and a CRNA (CJ) reviewed these tools to provide feedback and clarification and support iterative model development and validation. Anesthesia providers were given versions of the new tools developed and iteratively updated them to depict the nature of their work more accurately. This study did not utilize the systems story SEIPS 101 tool as this tool focused on the patients’ experiences and their story which is variable across different procedures, and while generally anesthetized, the patients are unaware of the medication delivery system (Holden and Carayon, 2021).

## 3. Results

### 3.1. Anesthesia PETT Scan (people, environment, tools, and tasks)

*The Anesthesia PETT Scan (People, Environment, Tools, and Tasks)* (Table 1) identifies key facilitators and barriers within a work system, grouping them into people, environment, tools, tasks, and their interactions between these system components. Facilitators are those systems features that encourage a quick process of anesthesia medication delivery, while barriers delay the process or increase the risk by adding complexity, extra steps, time, or uncertainty to the process (Holden and Carayon, 2021).

Effective teamwork and communication were identified as key people-related facilitators (Table 1), with barriers associated with differences in individual expertise, preferences, and potential conflict between providers. Negative relationships may arise due to clashing personalities, perspectives, professional conflicts, or an unwillingness to help one another. Physical facilitators included the visibility of the devices, team members, and surgical field, while the barriers included cramped workspaces and disorganization of tangled power cords, devices, and lines. Organizational environment facilitators included procedures, policies, and the OR social and safety culture. For instance, organizational encouragement of timeouts promotes a shared understanding of the operative plan (Berlinger and Dietz, 2016). Notably, anesthesia providers are prohibited by law from leaving medication unattended, resulting in frequent storing, and securing medication tasks upon exiting OR. Tool-related facilitators included phones providing direct lines of communication between staff, allowing for efficient and timely communication without forcing anesthesia providers to leave one patient to attend to another patient in a different room. Barriers found were primarily stemming from equipment issues (e.g., anesthesia providers having to troubleshoot malfunctioning equipment). No task-related facilitators were identified in this study; however, the buildup and accumulation of concurrent multiple tasks was a remarkable barrier.

The OR environment, such as the setup, noise level, and flow disruptions and task interruptions contribute to cognitive workload of the anesthesia provider. Interactions between the PETT factors that facilitated anesthesia work were noted at times when providers (people) sought advice from other anesthesia providers (people), whether that was in person or online (tools) via access to literature. Barriers to successful interactions included coordination and workspace issues related to other providers. For example, when team members (people) left the anesthesia workspace disorganized (environment), the next provider (people) was forced to clean up the workspace from the last procedure to prepare for the next procedures.

### 3.2. Anesthesia People Map

**Fig. 1.**
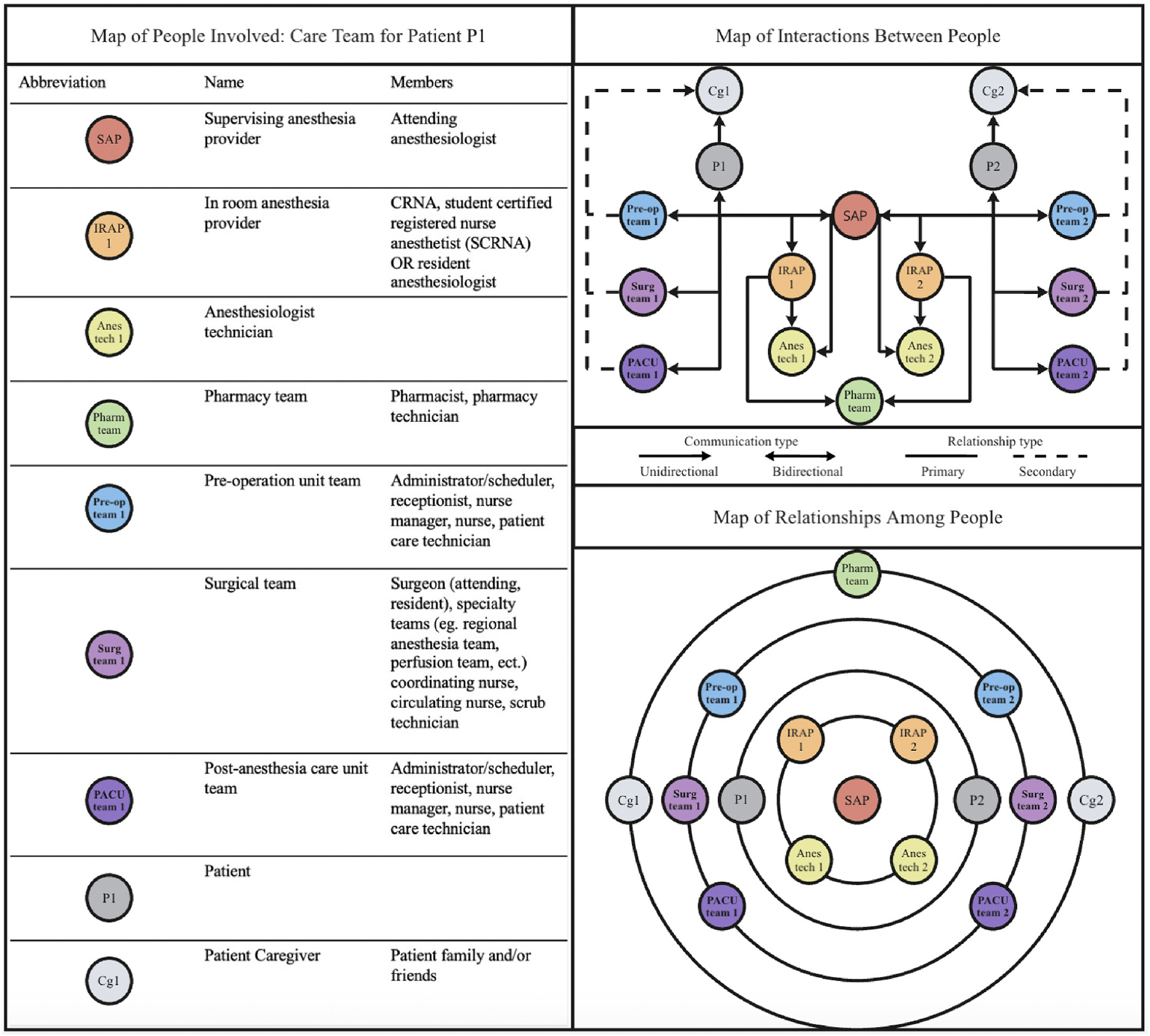
Anesthesia People Map. Circles represent team members and lines represent interactions and relationships.

*The Anesthesia People Map* (Fig. 1) includes a *Map of People Involved*, which describes the individuals and anesthesia care team members. *The Anesthesia People Map* also includes a *Map of Interactions Between People and the Map of Relationships Among People,* which describe the interactions between these individuals and their professional relationships to concentrically document their proximity to each other and the procedure. Though usually a people map would show individuals, it was necessary to merge some roles or responsibilities in order to represent the complexity of the work while remaining understandable.

Attending anesthesiologists care for up to four patients at one time, though only two patients are illustrated in the simplified figure presented here. They have the responsibility of establishing and maintaining work relationships with many other teams, who they may or may not have worked with before, including multiple CRNAs, resident anesthesiologists, anesthesia technicians, patients, patient families, surgical staff members, nurses, scrub technicians, pharmacists, pharmacy technicians, pre-operation unit (pre-op) staff, and post-anesthetic care unit (PACU) staff. Each care team member role has specific responsibilities that are necessary to successfully complete a surgical procedure, which requires a range of planned and ad-hoc interactions. Patient information typically flows back and forth between the anesthesiologist, the CRNA or resident, and the anesthesia technician. Ongoing interactions between multiple providers demonstrate how anesthesia providers continuously rely on the responses and actions of others to provide effective patient care.

### 3.3. Anesthesia Task x Tools Matrix

**Fig. 2.**
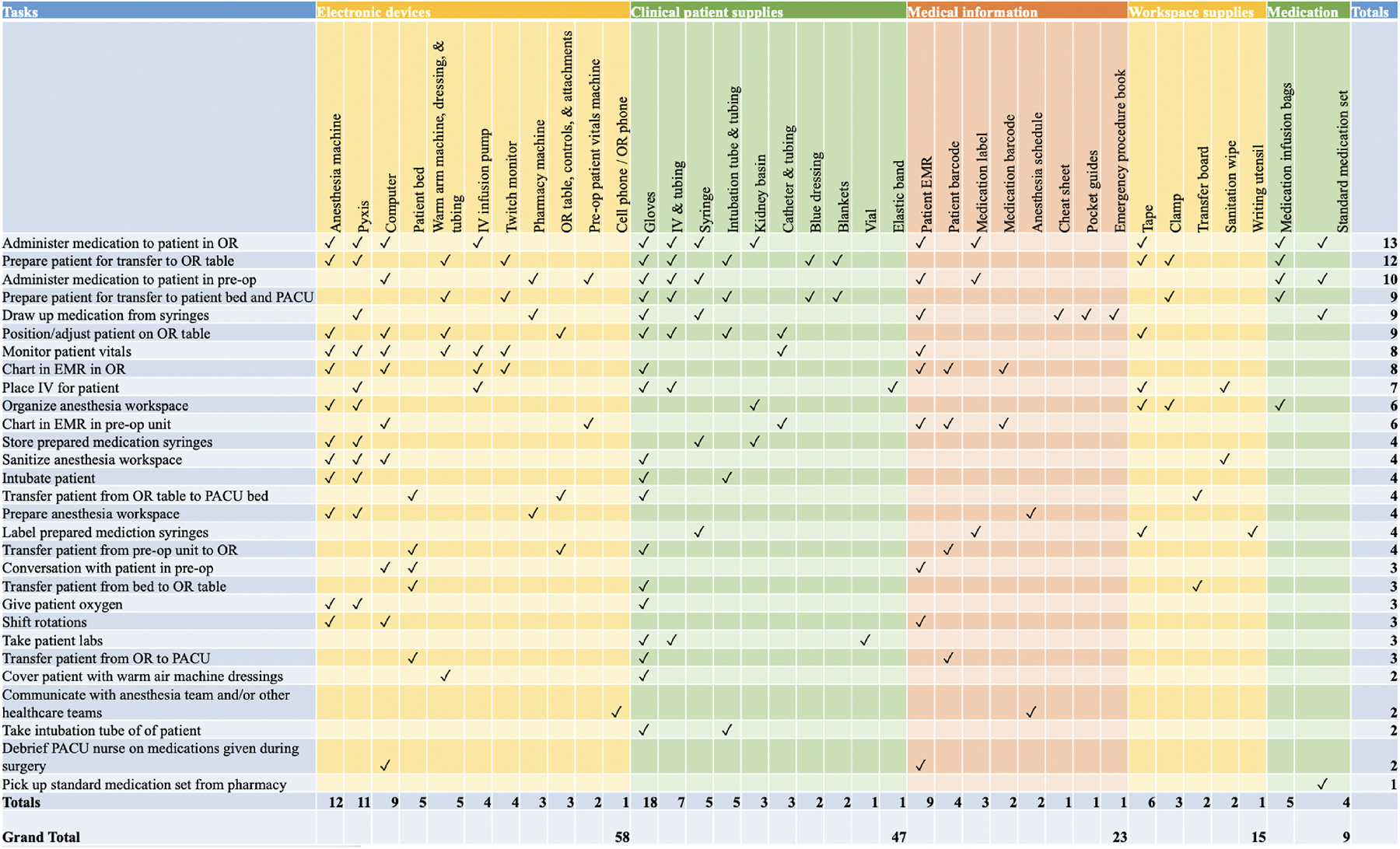
Anesthesia Task x Tools Matrix. Check marks indicate when a tool when required for a task during a case.

*The Anesthesia Task x Tools Matrix* (Fig. 2) identifies the key tasks demonstrated by the anesthesia team during the surgical process and the tools used to accomplish the tasks. The tools were organized into categories that represent similar functionality.

There were 11 electronic devices, 10 types of clinical supplies, 8 sources of information, 5 types of workspace supplies, and two sources of medications. Within these categories, some tools were used more than others across different tasks. The gloves, anesthesia machine, and Pyxis medication stage cabinet were the most used. Cell phones, vials, elastic bands, cheat sheet pocket guides, emergency procedure books, and writing utensils were all used less frequently. The number of tasks that use each tool conveys the interconnectedness and level of dependency providers might have for the tool as well as the large number of tools needed to perform the work.

### 3.4. Anesthesia Journey Map

**Fig. 3.**
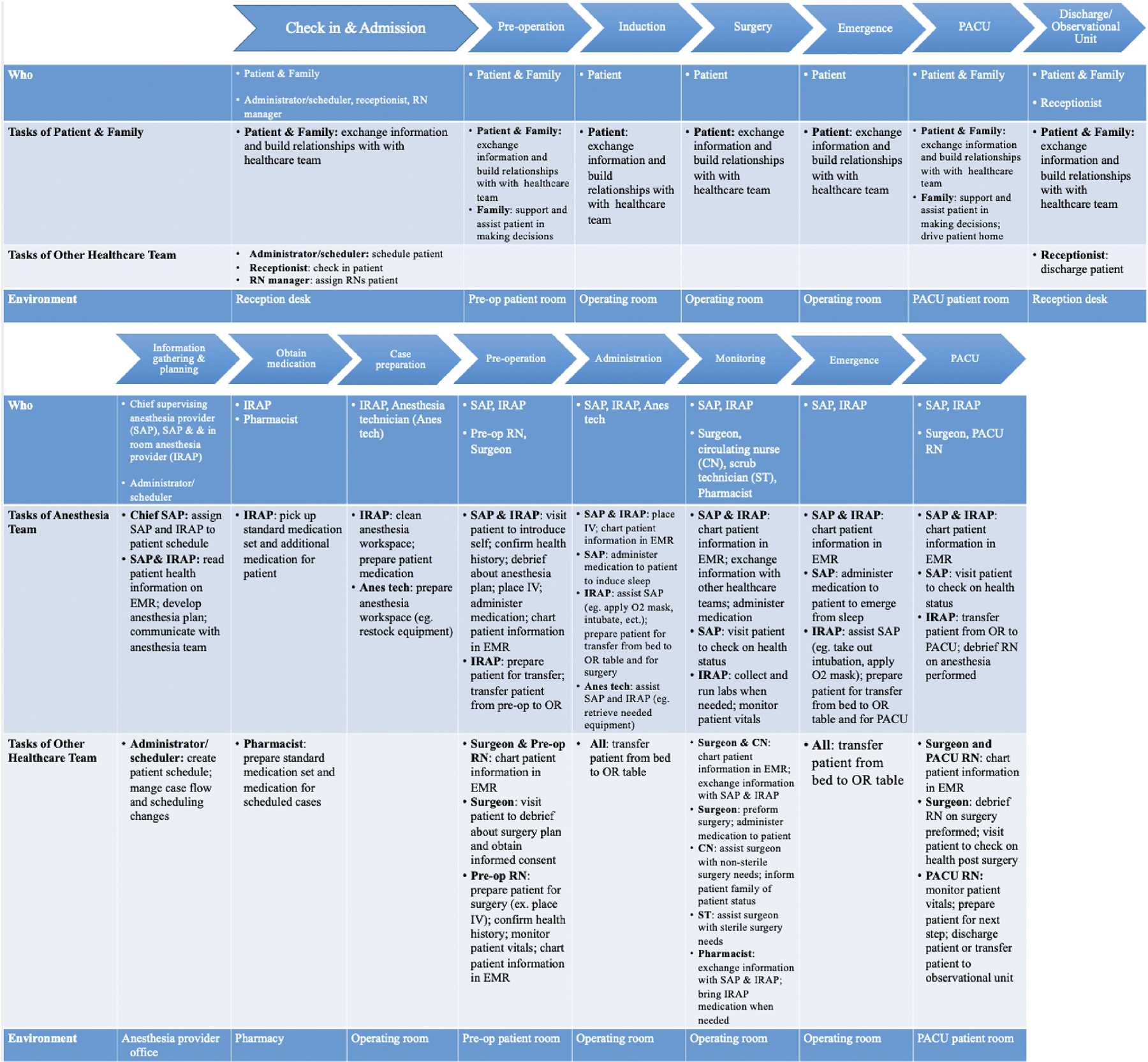
Anesthesia journey map.

*The Anesthesia Journey Map* (Fig. 3) demonstrates the patient and anesthesia team journey throughout a surgical process. Each column was used to represent an individual step of the journey, with the patient and anesthesia team aligned chronologically.

First, the anesthesia care team completes information gathering and planning before obtaining any medications. Both the patient and anesthesia team undergo induction, surgery, emergence, and post procedure care in the PACU together. Each step in the process requires different roles, tasks, and locations, while each role has different responsibilities, expertise which are not always carried out in the same manner, as these processes are subject to different peoples’ personal and professional preferences. Although they are working together, the supervising and in-room anesthesia providers may have different preferences for medication delivery. Also, different locations may have various tools, layouts, and supplies requiring atypical approaches and uncommon expertise (knowledge and skills not necessarily taught but learned) to carry out the work needed for caring for the patient at hand, as standardization is not always possible and may be restrictive needing adaptation in areas that may need improvement from redesign.

### 3.5. Anesthesia Work System Interactions Map

**Fig. 4.**
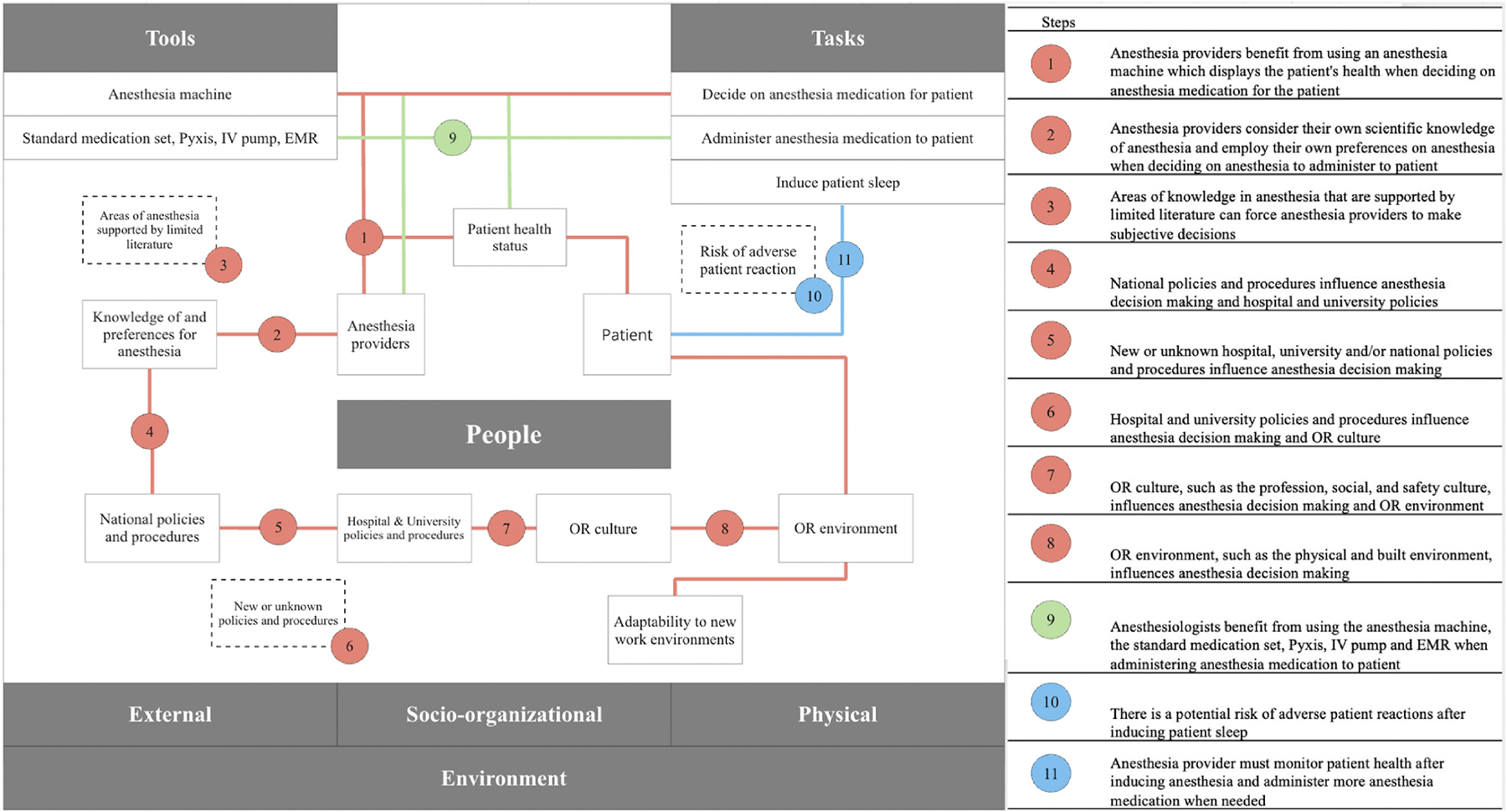
Anesthesia Work System Interactions Map. Red represents the anesthesia provider and the patient’s interactions, green represents the anesthesia provider’s interactions, and blue represents the patient’s interactions. The solid line boxes are tangible interactions, and the dotted boxes are theoretical interactions. (For interpretation of the references to color in this figure legend, the reader is referred to the Web version of this article.)

*The Anesthesia Work System Interactions Map* (Fig. 4) identifies essential and important interactions within the system of work that must occur between people, environment, tools, and tasks for successful medication delivery.

Fig. 4 identifies 11 key interactions that are necessary for successful medication delivery. The anesthesia provider (people) interacts with anesthesia machine (tool) to determine the patient’s (people) health status. Then, the anesthesia provider must use that information along with their own personal and professional knowledge and preferences of anesthesia to decide on the proper medication to administer to the patient. However, the anesthesia provider’s (people) own knowledge of anesthesia can be limited when and if there is little literature published or available on a topic, which can affect decision making and selection of the proper medication to administer to the patient. Interactions between work system factors can potentially conflict and lead to uncertainty (Carayon, 2012; Institute of Medicine Committee on Quality of Health Care inAmerica et al., 2000). For instance, at an academic medical center, national (e.g., Joint Commission), hospital, and university policies may not align. Misalignment of policies and guidelines forces anesthesia providers to make decisions on what policies and rules to follow. Consequently, anesthesia providers must account for and balance all these interactions when delivering care and administering medication, requiring knowledge, and learned experience for clinical decision making and balancing tradeoffs for the most ideal situation.

### 3.6. Anesthesia outcome matrix

*The Anesthesia Outcomes Matrix* (Table 2) demonstrates the desirable and undesirable outcomes that occur proximally and distally for the patient, family, provider, and organization. Proximal outcomes occur immediately after medication delivery and surgery, while distal outcomes occur sometime later.

Each stakeholder experiences different outcomes that are focused on different goals and people (Table 2). The patient’s outcomes are more focused on their health and well-being. The anesthesia provider’s outcomes are focused on successful and safe administration of medication without medication errors, quality of work life, and efficiency. Organizational outcomes include performance evaluations and earnings. Multiple outcomes contribute to the overall system complexity of anesthesia care delivery as they may not align across entities. We did not consider the outcome of the surgery specifically here, which may not relate to the quality of the anesthesia delivery. We also assumed that patients were not specifically concerned with errors, and indeed would most likely be unaware of them, unless they had a noticeable effect on their outcome.

## 4. Discussion

The application of the SEIPS 101 tools reveals the anesthesia medication delivery system to be complex and non-linear, involving multiple people, devices, information sources, locations, and outcomes. Typically, six to eight care team members are involved in medication administration for every two patients. Completion of the key assessment, diagnostic, preparation, delivery, and monitoring tasks requires the coordination and integration of multiple electronic devices, supplies, information, and medications. Multiple patients, family, personal, professional, organizational, and regulatory outcomes need to be met (Carayon et al., 2006; Holden and Carayon, 2021; Holden et al., 2013). Patient interactions, specific teamwork activities, and technologies that aid in provider decision making and medication delivery act as facilitators. Barriers usually stem from the broader work context, such as: lack of familiarity with workspace, providers, or devices; unwillingness to collaborate; uncertainty, time pressures, workspace design, and device functionality. SEIPS captures key work system factors and their essential interactions to provide a safe and effective system, in the case, the healthcare system.

The tools were relatively easily deployed, and their format is useful for illuminating the complexity of anesthesia medication safety, which may not always be well acknowledged. Many factors that can affect performance are outside the control of any one individual. There is also no single “right way” to balance the many different outcomes possible, given varying patient, team, and work system contexts. Finally, achieving the best outcomes within an imperfect system may often require workarounds or non-evidence-based strategies (Jeffcott et al., 2009; Berlinger, 2016). Thus, the clinical expertise required to deliver medications safely is not just related to traditional clinical skills but also is dependent upon the ability to adapt to variations in teams, tasks, technologies, processes, and desired outcomes. This confirms the long held “systems view” of safety, which states that failures cannot only be the responsibility of one person, or related to one root cause, but should be seen as a failure to control for, and then adapt to, the inherent uncertainty and complexity of the work (Hollnagel et al., 2006; Hollnagel et al., 2015; Dekker, 2014). This creates new opportunities for improving the safety and efficiency of medication delivery, while also explaining how some interventions have experienced mixed results.

The application of these tools may have profound implications for our understanding of medication harms. Humans are usually the last line of defense against harm, so they are often seen as the “cause” of errors. We can firmly debunk the idea that success or failure can be solely attributable to one individual, event, or action. The validity of causational attributions such as “situational awareness failure” and “cognitive bias” are thus brought into question (Lusk et al., 2022; Douros, 2021). Furthermore, proximal patient outcomes alone (quality of care, length of stay, pain management) are not sufficient to understand a comprehensive view of success or failure, or to evaluate interventions upon. Patient centered care requires adaptation to a range of patient, surgical, team, and organizational contexts. This demonstrates not only the importance of considering personal, professional, regulatory, financial, and other organizational requirements in medication safety; but also provides methods for understanding their influences on individual behaviors. Rather than seeing medication harms purely as an error in hindsight, they could be seen as a failure to adapt to new circumstances or appropriately trade-off one outcome for another. Viewing anesthesia medication delivery as a linear deterministic process that can, and should be, wholly standardized provides a limited perspective on the causes of and solutions to failures. While in agreement with other theoretical approaches (Staender, 2015), our analysis is one of the first attempts to build the empirical evidence from observations and interviews. There is no “one right way” to deliver anesthesia medications.

The SEIPS 101 tools offer a valuable perspective on how and why some interventions might have produced mixed results. Adapting to multiple factors and outcomes means that some interventions come at the expense of other factors and outcomes, often in the form of short-cuts. For example, while barcode scanning might lead to safer intravenous drug administration, it can increase interactions with devices, or other tasks (Nanji, 2020; Wahr et al., 2017), increasing workload (Khan et al., 2016), eventually leading to short-cuts which create new paths for adverse events (Patterson et al., 2006). Additionally, while some standardization (of devices, processes, workspaces) could be advantageous, there is also no “one right way” that fits every anesthesia medication delivery scenario (Nanji, 2020; Wahr et al., 2017; Martin et al., 2017). Standard color-coded syringe labels or icons that distinguish between classes of drugs have been suggested to potentially lead to an increase in syringe swaps between drug classes due to newly developed short-cuts (Merry and Anderson, 2011; Webster et al., 2001). Common teamwork and communication interventions, such as closed-loop communication, double checks or formalized communication channels would benefit from a broader range of stakeholders, including patients, families and supporting roles (Martin et al., 2017; Samost- Williams and Nanji, 2020). Since communication with other team members and coordination of people, tasks and technologies attempts to standardize behaviors that are required for the necessary adaptation, they may be rejected or lead to undesirable outcomes on other dimensions (e.g., increasing costs or professional dissatisfaction).

Our results suggest that medication delivery requires frequent adaptation; therefore, interventions would benefit from a focus on helping clinicians understand when and how best to adapt to new situations. Also, different locations may have various tools, layouts, and supplies requiring atypical approaches and uncommon expertise (knowledge and skills not necessarily taught but learned) to carry out the work needed for caring for the patient at hand., as standardization is not always possible and may be restrictive needing adaptation in areas that may need improvement from redesign. Though adaptions are often seen as “violations” in the context of an adverse outcome, instead there should be opportunities to promote an open discussion to understand why violations were made as an adaption or tradeoff of work system factors and outcomes (Leahy et al., 2018; Dekker, 2014). It suggests that instead of standardizing behaviors, it is beneficial to control for unnecessary (systemic) variation, while enhancing necessary variations in human behavior. Achieving this involves enhancing four principles: monitoring, expectation, response, and learning (Hollnagel et al., 2006), with the reduction in complexity and workload also important principles.

Future interventions could consider multifunctional devices that reduce the number of tools used, and ultimately reduce the complexity of the system. For instance, storing all medical information in one place, such as in anesthesia information management systems, which combines record-keeping, management, quality assurance and research functions into one device (Ehrenfeld and Rehman, 2011), increasing quality of care and reducing workload (Ehrenfeld and Rehman, 2011). This analysis also has implications for clinical decision making and support, as these tend to focus on a small range of patient outcomes, rather than the process of how clinicians make decisions, where they go for their information, what information they use, how they weigh it, and how they enact those decisions, or even how the “best evidence” can be made available to them when needed.

Healthcare professionals, organizations, systems, and the patient safety movement have arguably adopted simplistic approaches to understanding and addressing clinical risk (Wears and Sutcliffe, 2019). This lack of systems engineering thinking and analysis is damaging to patients and professionals alike (Lusk et al., 2022). So even though these general findings – that systems are complex, adaptive, and benefit from analysis of the success of everyday work – may not be especially novel to a human factors and ergonomics audience, this work is still relatively novel in healthcare and especially in anesthesia medication delivery. We found that the SEIPS tools are relatively easy to apply to clinical work and provide perspectives on medication delivery that are largely absent from the narratives around medication error or harm (Biro et al., 2022), but feel that clinicians would benefit from preparation in systems engineering principles, or collaboration with human factors professionals during their deployment. This work also reflects the benefits of access to and work at the frontline of clinical care that has traditionally been challenging for human factors professionals, and the benefits of close collaboration between systems engineers and clinicians (Catchpole et al., 2021; Perry et al., 2021). We conclude that the SEIPS 101 tools, when deployed within a multidisciplinary team of human factors and specialist healthcare experts establishes a shared understanding of the complexity of medication delivery that helps moving away from a focus on error to a greater understanding of how complex clinical systems function.

## 5. Limitations

In exposing a deeper complexity within the medication delivery system, the SEIPS 101 tools both require more time, and greater systems analysis expertise than is usually available to frontline clinicians. Just as one strength of this SEIPS analysis is the multiple tools that are used, there may be other perspectives not developed here that would help to develop a greater understanding of medication delivery solutions. There is also a possibly of bias within our methodology. For instance, observer bias could be present due to the presence of the trained observers. Selection bias might also be present because of the use of convenience sampling. Additionally, as observations and interviews were conducted at a single institution, future work should examine the generalizability of this model to other institutions. Anesthesia care varies across institutions in terms of institutional culture, staffing models, and resource availability. For instance, smaller or resource-constrained hospitals might experience unique challenges not captured in this academic hospital setting. The SEIPS 101 tools presented here provide a basic model of patient care that can be easily adapted to represent the variations in practice. Prior to the development of interventions at specific institutions, the tools should be tailored to the specific workflow representative of that clinical system. Although a wide range of procedures were sampled, examining a wider range of surgical procedures with varying patient characteristics and type of anesthesia needed may also reveal other tasks, processes, and hazards.

## 6. Conclusion

The SEIPS 101 tools demonstrate the need to consider a broader range of factors and multiple important outcomes when developing successful interventions for perioperative medication delivery. There is no “one right way” to model a system, but the SEIPS 101 tools reveal a broader range of potential methods for understanding medication delivery beyond the more traditional approaches such as barcodes and standardization. Systems analyses also bring attention to and may potentially increase awareness of the limitations and pitfalls of current approaches and improve upon methods and interventions aimed at understanding errors, safety, and the nature of clinical expertise and decision making. Future studies should expand research on the systematic process of anesthesia medication delivery and on developing, testing, and implementing possible interventions that reduce complexity and workload while enhancing monitoring, expectations, response, and learning.

## Figures and Tables

**Table 1:** Anesthesia PETT scan (people, environment, tools, and tasks).

SEIPS Factors	Facilitators	Barriers

**People:** Patients	• Discussions with patients reveal history, comorbidities, allergies, and preferences that may not be documented	• Patient often not familiar with the surgical process so operating room (OR) staff must inform them and ease their fears • Patient often unaware of anesthesia risks until informed by anesthesia team in preoperative unit so patient must determine consent quickly
**People:** Healthcare Professionals	• Anesthesia team displays teamwork during induction • All OR staff help transfer patient from the patient bed to OR table and back	• Anesthesia providers vary in decision making and treatment strategies which may lead to team confusion • Indefinite/uncommon areas in anesthesia require subjective decision making • Anesthesia team must wait on the surgeon to talk to patient in preoperative unit
**People:** Others		• Family often uncertain of patient status while patient in surgery because they are only updated every few hours by circulating nurse
**Environment:** Physical	• Streamlining tasks for each case allows anesthesia team to better adapt to new environments (quicker/easier orientation/setup) • Visibility of monitors, team, and the surgical field aids awareness	• Cords from machines and infusion intravenous (IV) lines used by anesthesia team are often in the way of tasks and/or require untangling • In-room provider must unhook patient from all monitors every time they need to transfer or reposition patient • Anesthesia area varies in size and shape for each location so anesthesia team must adapt • Low lighting in OR can lead to difficulty reading medication labels
**Environment:** Socio-organizational	• Timeout ensures OR staff understands plan for procedure • Overall collaborative culture in OR because everyone helps with tasks	
**Environment:** External		• Anesthesia team cannot leave medications out unattended
**Tools**	• Anesthesia machine displays all vitals and automatically transfers them to patient electronic medical record (EMR) chart • IV pump gives patient medication at a set rate • Phones allow in-room and supervising provider communicate about patient instantly and from separate areas	• OR table remote has short cord at top near anesthesia area so inroom provider must control all bed adjustments • Preoperative unit bed often not fully charged which can cause difficultly for in-room provider to move it • OR table remote often does not work properly so in-room provider must troubleshoot • Anesthesia team uses many machines with many drawers so must constantly pull drawers in and out • IV pump requires tedious steps to program settings for each medication • Limited access to computer to chart patient information on EMR • Not all sharps fit into sharp disposal so anesthesia team must place on top of disposal
**Tasks**		• Anesthesia team must perform most tasks at induction • In-room provider prepares and labels all medication at same time • In-room provider rotations can lead to confusion because new provider unsure of where everything (eg. medication) was placed by previous provider • Supervising provider must juggle multiple patients at one time and choose how to allot time to each
**Interactions Between People, Environment, Tools, and Tasks**	• Anesthesia team able to consult other Anesthesia team colleagues (people) and literature on computer (tools) on patient decisions	• Anesthesia area (environment) left messy from anesthesia provider from day before (people) so anesthesia provider must clean before every case • Anesthesia technicians (people) often put equipment (tools) on incorrectly (eg. backwards) and leaves cords tangled so anesthesia provider must fix before every case • OR staff inputting in EMR (tool) can omit important information which can confuse other OR staff (people) because staff heavily rely on it for information • In-room provider must control temperature of room (environment) for patient, but must rely on circulating nurse (people) to turn up/down air conditioning

**Table 2:** Anesthesia outcomes matrix.

		Potential Outcomes			
		Patient	Family	Anesthesia Providers	Organization

**Proximal**	**Desirable**	• Patient experiences increased (improved) physical, mental, and emotional health due to anesthesia • Patient experiences decreased burden and stress due to anesthesia • Patient is satisfied with anesthesia care provided	• Family experiences increased (improved) mental and emotional health due to improved patient health • Family experiences decreased caregiver burden and stress	• Anesthesia administers anesthesia to patient without medication errors • Anesthesia experiences increased confidence in self to provide anesthesia • Anesthesia maintains occupation	• Organization performance increases • Organization experiences short term earnings • Organization maintains staffing
	**Undesirable**	• Patient experiences decreased physical, mental, and emotional health and/or death due to anesthesia • Patient experiences increased burden and stress due to anesthesia • Patient is not satisfied with anesthesia care provided	• Family experiences decreased mental and emotional health due to decreased patient health • Family experiences increased caregiver burden and stress	• Anesthesia administers anesthesia to patient with medication errors • Anesthesia experiences decreased confidence in self to provide anesthesia • Anesthesia is fired	• Organization performance decreases • Organization experiences decreased staffing
**Distal**	**Desirable**	• Positive longitudinal health outcome • Patient experiences increased trust in anesthesia provider	• Family experiences increased trust in healthcare provider	• Anesthesia experiences increased efficiency of delivering anesthesia • Anesthesia experiences increased quality of work life (ex: job satisfaction, increased engagement)	• Organization experiences increased public confidence in healthcare delivery (positive press) • Organization experiences an increase in turnover of anesthesia cases • Organization experiences long term earnings
	**Undesirable**	• Negative longitudinal health outcome • Patient experiences decreased trust in anesthesia provider • Financial burden from cost of anesthesia	• Family experiences decreased trust in healthcare provider • Financial burden from cost of anesthesia	• Anesthesia is served with a medical lawsuit • Anesthesia experiences decreased efficiency of delivering anesthesia • Anesthesia experiences decreased quality of work life (ex: burnout, decreased engagement)	• Organization served medical lawsuit • Organization experiences decreased public confidence/negative press • Organization experiences decreased case turnover • Organization experiences new laws/patient safety regulations • Organization experiences long term loses

## Abbreviations

FMEA: Failure Modes Effects Analysis
STPA: Systems-Theoretic Process Analysis
SEIPS: Systems Engineering Initiative for Patient Safety
STPA: Systems-Theoretic Process Analysis
PETT: People, Environment, Tools, and Tasks
OR: Operating room
CRNA: certified registered nurse anesthetists
IRAP: in-room anesthesia providers
SAP: supervising anesthesia providers
IV: infusion intravenous
EMR: electronic medical record
Pre-op: pre-operation unit
PACU: post-anesthetic care unit
